# Targeting β-glucans, vital components of the *Pneumocystis* cell wall

**DOI:** 10.3389/fimmu.2023.1094464

**Published:** 2023-02-09

**Authors:** Mengyan Wang, Zhongdong Zhang, Xiaotian Dong, Biao Zhu

**Affiliations:** ^1^ Department II of Infectious Diseases, Xixi Hospital of Hangzhou, Hangzhou, China; ^2^ Department of Infectious Diseases, State Key Laboratory for Diagnosis and Treatment of Infectious Diseases, Collaborative Innovation Center for Diagnosis and Treatment of Infectious Diseases, National Clinical Research Center for Infectious Diseases, The First Affiliated Hospital, College of Medicine, Zhejiang University, Hangzhou, Zhejiang, China; ^3^ Department of Clinical Laboratory, The First Affiliated Hospital, College of Medicine, Zhejiang University, Hangzhou, Zhejiang, China

**Keywords:** *Pneumocystis*, β-glucan, inflammatory, initial immunity, therapy

## Abstract

β-glucan is the most abundant polysaccharide in the cell wall of *Pneumocystis jirovecii*, which has attracted extensive attention because of its unique immunobiological characteristics. β-glucan binds to various cell surface receptors, which produces an inflammatory response and accounts for its immune effects. A deeper comprehension of the processes by *Pneumocystis* β-glucan recognizes its receptors, activates related signaling pathways, and regulates immunity as required. Such understanding will provide a basis for developing new therapies against *Pneumocystis*. Herein, we briefly review the structural composition of β-glucans as a vital component of the *Pneumocystis* cell wall, the host immunity mediated by β-glucans after their recognition, and discuss opportunities for the development of new strategies to combat *Pneumocystis*.

## 1 Introduction

The ascomycetous fungus, *Pneumocystis jirovecii*, is the causative agent of serious fungal pneumonia, predominantly occurring in immunocompromised individuals, especially HIV-positive patients. The use of antiretrovirals and medications for prophylaxis in the developed world has led to a decline in the mortality of *Pneumocystis* pneumonia (PCP) in patients with HIV in recent years. However, *Pneumocystis* remains an important pathogen worldwide because of the continued epidemic of acquired immunodeficiency syndrome (AIDS). *Pneumocystis* is also a common pathogen in non-HIV individuals taking immunosuppressive medications. PCP has high mortality and morbidity, which are commonly higher in patients without AIDS than in those with AIDS (1). The mortality of PCP is 10%–30% in AIDS patients and 40%–70% in non-AIDS patients (2–5). This difference may be explained by the greater lung damage in non-AIDS patients caused by intense lung inflammation. Thus, research should focus on finding better therapies for PCP.

## 2 Cell wall structure

*Pneumocystis* has a high tropism for the lung and is usually only detected in the lungs of infected hosts, where it completes all of its life cycle stages (6). The cell wall of *Pneumocystis* comprises β-glucans, chitins, and other carbohydrate polymers as a dynamic carbohydrate backbone (7, 8). This dynamic carbohydrate backbone plays an important role in the integrity and growth of *Pneumocystis* and mediates the immune response of the host to *Pneumocystis*. The cell wall components of *Pneumocystis* vary at different stages of the life cycle, e.g., the cystic and trophic forms. *Pneumocystis jirovecii* has three distinct life cycle stages (trophic form, pre-cystic form, and mature cyst), which have different structures and sizes while growing in the lung (6). The pre-cystic and trophic forms lack β-glucans (9). A recent study considered that the *Pneumocystis* cell wall lacks chitin, outer chain N-mannans, and α-glucan, which are present in many other fungi (10).

β-glucans represent important components of the *Pneumocystis* cell wall. Fungal cell wall β-glucans comprise a β-1,3-glucan backbone with variable side chain β-1,6 linkages (11). Many of the enzymes located within the cell membrane participate in the formation of the glucose backbone of the *Pneumocystis* cell wall (**Table 1**). The enzyme PcGsc-1 (glucan synthetase) forms the essential β-1,3-glucan backbone by polymerizing uridine-5-diphosphoglucose (12). PcGsc-1 is activated by PcAce2, which is phosphorylated by the upstream PcCbk1, a cell wall biosynthesis kinase (18). A recent study proposed that *Pckre6* encodes β-1,6-glucan synthase in *Pneumocystis* (13). *Pneumocystis Pcphr1* is believed to encode a protein that binds to the β-1,6-glucans of the β-1,3 backbone (14), and it is also pH responsive, which helps the cell wall to adapt to changing environmental conditions. β-1,3 endoglucanase has the most important role of degrading β-glucans in fungal cell walls. The single copy gene, *Pceng2*, encodes an endo-β-1,3-glucanase (15), which probably functions to switch the expressed major surface glycoprotein (Msg) variant (16). Bgl2 is considered to have an endo-β-1,3-glucanase activity and a glucanosyltransferase activity in *Pneumocystis*, and it exerts this glucanosyltransferase activity by cleaving reduced laminaripentaose and transferring oligosaccharides, resulting in polymers of six and seven glucan residues (17). In addition, β-1,3-glucan in the host serum has a critical role in the diagnosis of PCP; however, novel systematic reviews and meta-analyses have been reported. They found that sensitivity was 91% and specificity was 79% for the diagnosis of PCP. The sensitivity was better in HIV patients (94%) than that in non-HIV patients (86%), and the specificity was equivalent (83% vs. 83%) (19). Here, we do not describe the diagnostic value of β-1,3-glucan in detail again. *Pneumocystis* glucan provides cell wall stability and mediates the host lung inflammatory and immune responses.

**Table 1:** Genes encode enzymes located within the cell membrane that participate in the formation of the glucose backbone in the *Pneumocystis* cell wall.

Gene	Function of the encoded protein	Reference
*PcGsc-1*	Polymerizes β-1,3-glucan	(12)
*Pckre6*	β-1,6-glucan synthase	(13)
*Pcphr1*	Encodes a protein that binds β-1,6-glucans to the β-1,3 backbone and is pH responsive	(14)
*Pceng2*	Encodes endo-β-1,3-glucanase and switches the expressed major surface glycoprotein (Msg) variant	(15, 16)
*Bgl2*	Endo-β-1,3-glucanase and glucanosyltransferase	(17)
*PcAce2*	Activates PcGsc-1	(18)

Toll-like receptors (TLRs); Ephrin type-A receptor 2(EphA2).

## 3 Immune recognition

**Figure 1:**
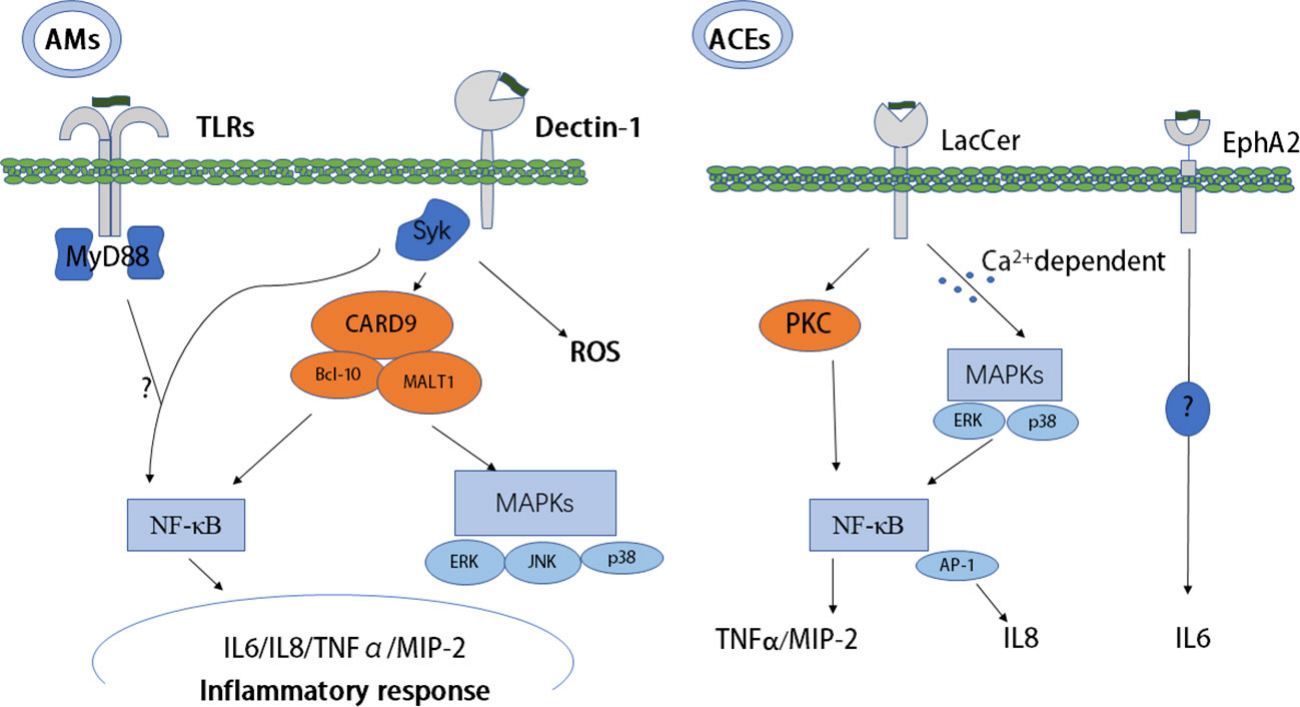
Host recognition of PCBG and PCBG-mediated initial immunity. Dectin-1, TLRs, EphA2, and LacCer as receptors recognize PCBG and mediate initial immunity resulting in an inflammatory response. Collaboration between dectin-1 and TLRs to mediate the NF-κB pathway is unclear, and the signaling pathway of EphA2 is also very unclear. AMs, alveolar macrophages; MyD88, myeloid differentiation primary response 88; NF-κB, nuclear factor kappa-light-chain-enhancer of activated B cells; PC, *Pneumocystis*; SYK, spleen tyrosine kinase; TLRs, Toll-like receptors; EphA2, ephrin type-A receptor 2; MIP-2, macrophage inflammatory protein 2, TNF-α, tumor necrosis factor-alpha; AECs, alveolar epithelial cells; PCBG, *Pneumocystis carinii* cell wall constituent β-1,3-glucan; LacCer, lactosylceramide.

The molecular immunogenic signatures of fungal pathogens in the cell wall are known as pathogen-associated molecular patterns (PAMPs). PAMPs usually comprise essential structural components that are lacking in the host and are recognized by host cells *via* binding to cellular or soluble pattern recognition receptors (PRRs). PRRs comprise four main types, RIG-I-like receptors (RLRs), NOD-like receptors (NLRs), C-type lectin-like receptors (CLRs), and Toll-like receptors (TLRs), which are expressed in most cell types (20). β-glucan, as a kind of PAMP, is important in the recognition of *Pneumocystis* by both alveolar macrophages (AMs) and alveolar epithelial cells (AECs). Glucans have a number of potential receptors, such as dectin-1 and TLRs (21). The major receptors of *Pneumocystis* β-glucans and the initial immunity mediated by β-glucan through these receptors are discussed below (**Table 2**; **Figure 1**).

**Table 2:** Host immune receptors and relevant cell types that express the receptors.

Receptor	Cell types	Reference
Dectin-1	Macrophages, dendritic cells (DCs), bronchial epithelial cells, pulmonary epithelium cells	(22–24)
TLRs	Macrophages	(25–27)
EphA2	Alveolar epithelial cells (AECs)	(28, 29)
Lactosylceramide	AECs	(30, 31)

Toll-like receptors (TLRs); Ephrin type-A receptor 2(EphA2).

### 3.1 Dectin-1

Studies have confirmed that the CLR dectin-1 is vital to recognize and kill *Pneumocystis* and is important in the production of proinflammatory mediators (32). The two main pathways of CLR signaling are the Ras-Raf pathway and the spleen tyrosine kinase (SYK) pathway. Dectin-1 recruits SYK directly or following binding with the Fc receptor c chain (FcRc) (33). After binding to β-glucan, dectin-1 activates SYK, leading to downstream inflammatory signaling (34). After recognizing *Pneumocystis*, dectin-1 induces internalization and killing, resulting in the production of reactive oxygen species (ROS) in AMs (22). In addition to AMs, dendritic cells (DCs) can also be activated by β-glucans, resulting in T-cell activation and polarization into a Type 1 T helper (Th1) patterned response, but with the absence of interleukin (IL)-12. Furthermore, DC activation is partially regulated by dectin-1 receptor-induced cytokine secretion and by Fas-FasL, which further promotes inflammation, resulting in tumor necrosis factor-alpha (TNF-α) and IL-1β secretion in PCP (23). Interestingly, in response to *Pneumocystis* β-glucan, dectin-1 does not affect TNF-α and IL-12 secretion (22). However, dectin-1 expression can enhance β-glucan–containing particle activation of nuclear factor kappa-light-chain-enhancer of activated B cells (NF-κB) mediated by TLRs. Subsequently, TLRs and dectin-1 synergistically regulate the production of IL-12 and TNF-α through myeloid differentiation primary response 88 (MyD88) in macrophages and DCs (22, 24).

### 3.2 Toll-like receptors (TLRs)

TLRs activate two main signaling cascades: the The toll-interleukin-1 receptor (TIR) domain-containing adapter-inducing Interferon-B (TRIF) pathway and the MyD88 pathway (35). Our understanding of the functions of TLRs in the immune response induced by *Pneumocystis* is incomplete. *Pneumocystis murina* activates TLR2 in AMs, resulting in NF-κB nuclear translocation and the production of the proinflammatory cytokine TNF-α and chemokine macrophage inflammatory protein 2 (MIP-2) (25). Deficiency in TLR2 increases the susceptibility to *P. murina*, and it was proposed that TLR2-mediated inflammatory responses can help to clear *Pneumocystis* in mice (36). However, TLR2 is required by AMs, but not by epithelial cells, to trigger an inflammatory response (26). MyD88-deficient mice demonstrated a partially blunted response of AMs to *Pneumocystis* β-glucan, suggesting that macrophages are activated through NF-κB *via* cellular receptors and signaling pathways (27). MyD88-deficient mice also demonstrated the role of MyD88 signaling in both immunopathogenesis and the control of the fungal burden (37). However, Ripamonti et al. (38) found that MyD88 signaling is not required to control *Pneumocystis* infection. Deficiency of either SYK or TLRs (TLR4, TLR5, TLR7, and TLR9), or the adapter MyD88, abolished the production of TNF, MIP-1α, and MIP-2. Therefore, collaboration between SYK pathway signaling, activated by dectin-1, and TLRs/MyD88 signaling pathways is required to enhance NF-κB nuclear translocation (39). Furthermore, in human primary peripheral blood mononuclear cells (PBMCs) and in monocyte-derived macrophages, collaboration between dectin-1 and TLRs (TLR2, TLR4) to produce TNF-α has been demonstrated (40).

### 3.3 Lactosylceramide

AECs play an important role in promoting *Pneumocystis carinii* attachment and mediate lung inflammation *via* the production of cytokines and chemokines in PCP. Lactosylceramide is a prominent cell membrane glycosphingolipid stimulated by *P. carinii* cell wall constituent β-1,3-glucan (PCBG), subsequently causing the release of MIP-2 from isolated AECs (30). In addition, the release of IL-8 requires glycosphingolipid for optimal signaling after activation of PCBG by mitogen-activated protein kinases (MAPKs) (31).

### 3.4 Ephrin type-A receptor 2

CLRs are critical in the myeloid cell response after recognizing *Pneumocystis*, as proven *in vitro* and *in vivo* (21). However, the inflammatory reaction has been proven to occur in epithelial cells, which release cytokines such as IL-6, IL-8, MIP-2, and TNF-α after recognizing fungal β-glucans (41). A previous study identified ephrin type-A receptor 2 (EphA2) as a receptor that binds to fungal β-glucans in lung epithelial cells (28). β-glucans isolated from exposed surfaces of *Pneumocystis* can also bind with EphA2. Furthermore, the EphA2 receptor can be phosphorylated after the binding, resulting in a downstream proinflammatory response and increased IL-6 cytokine production in lung epithelial cells (29).

## 4 β-glucan mediated initial immunity

The host’s innate immune system is activated greatly by *Pneumocystis* cell wall components, including β-glucans (42). However, *Pneumocystis* has evolved mechanisms to evade and adapt the host response.

AMs, as professional phagocytic cells, recognize microorganism surface PAMPs *via* PRRs. Attachment and activation of macrophages result in phagocytosis, followed by phagolysosomal fusion and degradation of the microorganisms. AMs are important because they can directly kill trophozoites and cysts, and the severity of PCP and the number of macrophages correlate inversely (43). PCBG from AMs activates NF-κB translocation, which stimulates the production of TNF-α and MIP-2, the murine homolog of IL-8 (27). Steele et al. (32) reported that after *Pneumocystis*–AM interaction, dectin-1 generates ROS, which mediates nonopsonic phagocytosis and subsequent killing of the pathogen. In *Pneumocystis* infection, knockout of dectin-1 in macrophages resulted in defective ROS production (22). The adapter molecule, caspase recruitment domain-containing protein 9 (CARD9), is initiated through dectin-1. Subsequent to SYK activation, CARD9 forms a trimolecular complex with B-cell lymphoma/leukemia 10 (BCL-10) and mucosa-associated lymphoid tissue lymphoma translocation protein 1 (MALT1), which activates NF-κB and MAPKs, such as p38, c-Jun N-terminal kinase (JNK), and extracellular signal-regulated kinase (ERK), resulting in the production of a proinflammatory response (44). Moreover, vitronectin and fibronectin binding to PCBG can augment macrophage inflammatory responses (45). Scott et al. proposed that in AECs, the cell surface lactosylceramide, rather than the dectin-1 receptor, mediated chemokine responses. In PCP, AECs are important in host responses, and the production of inflammatory cytokine is induced by β-glucan *via* NF-kB–dependent mechanisms, which are partly mediated by protein kinase C (PKC) signaling pathways (46). PKC localizes to AEC microdomains, which promote the expression of TNF-α and the rodent C-X-C chemokine MIP-2, in addition to identified inflammatory secondary signaling pathways (47). This might be a potential novel target for therapeutics in immunocompromised populations. Furthermore, PCBG stimulates DCs to interact with lymphocytes, resulting in the activation of the IL-23/IL-17 axis during infection, which functions *via* the accumulation of lactosylceramide in glycosphingolipid-rich microdomains of the plasma membrane (48). In addition, in a calcium-dependent manner, PCBG can also induce MAPK, ERK, and p38 phosphorylation, NF-κB activation, and subsequent IL-8 secretion through a possible receptor (glycosphingolipids) in human airway epithelial cells, resulting in neutrophil infiltration (31). Rapaka et al. (49) proposed that natural immunoglobulin M (IgM) modulates innate and adaptive immune responses by binding to *Pneumocystis* β-glucan. Furthermore, depletion of CD4+ T cells did not affect the amount of β-glucan cross-reactive IgG in the serum or mucosa but decreased lung mucosal levels of cross-reactive IgA at the same time as reducing active transforming growth factor β activation. Moreover, despite CD4+ T-cell depletion, IgM levels increased significantly. Thus, an immune response against *Pneumocystis* β-glucan might occur under conditions of CD4+ T cell-related immunodeficiency *via* differential CD4+ T cell-dependent regulation of mucosal antibody responses (50).

The specific PRR (dectin-1) for β-glucan has been discovered on the cell surface of phagocytes (51); therefore, β-glucan, as a key PAMP, has attracted increased attention in the study of the immune recognition of pathogenic fungi by the host. However, certain fungi have evolved surface structures that allow them to bypass this innate immune control mechanism. β-glucans of *Candida* and *Aspergillus* pathogens can be masked *via* a thick layer of mannoproteins, abrogating the activation of host innate immune responses (52, 53). Furthermore, Ballou et al. (54) showed that β-glucan on the *Candida* cell surface can be masked by L-lactate generated by host cells or bacteria from the host’s microbiota. This represents the immune escape of β-glucan masking in *Candida*, which reduces fungal visibility to the host immune system (54). In the *Pneumocystis* cyst stage, β-glucans are a major constituent of the cell wall, which can activate innate immune responses. Kutty et al. (55) demonstrated that *Pneumocystis* cyst cell wall β-1,3-glucans are largely masked by Msg and/or other surface proteins, which would likely block the activation of the innate immune response. Presumably, this mechanism evolved to adapt to immunocompetent hosts with reduced organism loads. Organism death and release of glucans might be important factors in deleterious host inflammatory responses in immunosuppressed hosts with a high organism burden (55). *Pneumocystis* Msg is a 120-kDa surface protein complex with important functions in adhesion and immune recognition. Kottom et al. (56) showed that the *Pneumocystis* Msg surface protein complex can suppress TNF-α secretion from macrophages induced by proinflammatory β-glucans.

## 5 Clinical therapeutic strategies and future perspectives

Currently, trimethoprim–sulfamethoxazole (TMP-SMX) is the first-line antibiotic to treat PCP (57). Pentamidine, clindamycin, primaquine, and atovaquone, as second-line agents, are reserved for mild to moderate disease (58). TMP-SMX can inhibit dihydropteroate synthase (DHPS) and impair the synthesis of folate in *Pneumocystis*. DHPS mutants in *Pneumocystis* have been reported, which might result in resistance to TMP-SMX therapy, and the reported increase in the frequency of DHPS mutations might be the result of selection pressure from using TMP-SMX for the prophylaxis and treatment of *Pneumocystis* globally (59). Echinocandin antibiotics, such as caspofungin, can be used to treat *Pneumocystis* infection, which disrupt the integrity of the cell wall by inhibiting β-1,3-glucan synthetases. Echinocandins are highly effective in eliminating the cyst forms in animal models but far less effective against the trophic forms (60). Our previous study proved the effectiveness of echinocandin treatment for mild to moderate AIDS-PCP disease (61). Therefore, it is crucial to develop novel therapies for *Pneumocystis* from different perspectives and to improve clinical outcomes and reduce the mortality of this deadly infection.

Respiratory failure associated with PCP resulting in intense lung inflammation is a major cause of death in immunocompromised patients. Moreover, β-glucan may be a potential target for the treatment of *Pneumocystis* infection from the aspect of destroying structures and inhibiting pathways, immunity, and inflammatory reactions. In steroid-treated infected rats, aculeacin A, a beta-1,3-glucan biosynthesis inhibitor, ameliorated *Pneumocystis* cell wall formation and cyst maturation, thereby preventing PCP (62). A recombinant protein, dectin-Fc, comprising dectin-1 fused to the Fc region of murine IgG1, could be used to specifically identify β-1,3 glucan linkages. Targeting *Pneumocystis* β-glucan with dectin-Fc enhanced host recognition and the clearance of *Pneumocystis* and might enhance resistance to PCP in immunodeficient hosts (63). Similarly, dectin immunoadhesins (dectin-1:mIgG1 and dectin-1:mIgG2a Fc), comprising dectin-1 fused to the Fc regions of the four subtypes of murine IgG (mIgG), could reduce cytokine production and hypoxemia, although they had less effect on the lung fungal burden (64). Furthermore, CARD9, as a downstream factor of dectin-1, is activated by recognition of β-glucans, resulting in damaging inflammation. A CARD9 inhibitor (BRD5529) was shown to reduce phospho-p38 and phospho-pERK1 signaling and TNF-α release during *Pneumocystis* β-glucan stimulation of macrophages (65). Macrophages can enhance effector functions toward subsequent heterologous stimuli after exposure to the fungal β-glucan as a kind of trained immunity. In addition, trained macrophages displayed increased glycolysis and oxidative phosphorylation when competing for limiting levels of nutrients (66). The mechanistic target of rapamycin (mTOR)/hypoxia-inducible factor 1 alpha (HIF-1α) axis regulates the functional and metabolic reprogramming of β-glucan-trained macrophages (67). Combining inhibitors of glucose uptake and glycolysis with antifungal agents, with the aim of decreasing inflammation and consequently diminishing the development of resistance to antifungals, might represent a potential therapeutic strategy.

## 6 Conclusion

Our understanding of *Pneumocystis* immunobiology has grown rapidly in recent decades. Vital components of the *Pneumocystis* cell wall and their synthetic mechanisms, as well as important interactions with the host, have been proposed. Comparisons with closely related fungi have revealed important cell signaling pathways in *Pneumocystis*. β-glucan has been demonstrated experimentally to have a vital function in the interaction between the host and the pathogen during infections. Research has revealed some molecular and cellular mechanisms by which β-glucan mediates immunity and immune escape in *Pneumocystis* infections. However, further studies to refine the immunomodulatory mechanisms and to develop therapeutic strategies are needed.

## Author contributions

Conceptualization, MW, ZZ, and BZ; Data curation, MW and XD; Software analysis, MW and XD; Original draft preparation, MW, ZZ, and BZ; Review and Editing, MW and BZ; Supervision, BZ; Project administration, BZ; Funding acquisition, MW and BZ. All authors contributed to the article and approved the submitted version.
